# Correlation between genomic index lesions and mpMRI and ^68^Ga-PSMA-PET/CT imaging features in primary prostate cancer

**DOI:** 10.1038/s41598-018-35058-3

**Published:** 2018-11-12

**Authors:** Claudia Kesch, Jan-Philipp Radtke, Axel Wintsche, Manuel Wiesenfarth, Mariska Luttje, Claudia Gasch, Svenja Dieffenbacher, Carine Pecqueux, Dogu Teber, Gencay Hatiboglu, Joanne Nyarangi-Dix, Tobias Simpfendörfer, Gita Schönberg, Antonia Dimitrakopoulou-Strauss, Martin Freitag, Anette Duensing, Carsten Grüllich, Dirk Jäger, Michael Götz, Niels Grabe, Michal-Ruth Schweiger, Sascha Pahernik, Sven Perner, Esther Herpel, Wilfried Roth, Kathrin Wieczorek, Klaus Maier-Hein, Jürgen Debus, Uwe Haberkorn, Frederik Giesel, Jörg Galle, Boris Hadaschik, Heinz-Peter Schlemmer, Markus Hohenfellner, David Bonekamp, Holger Sültmann, Stefan Duensing

**Affiliations:** 1 Department of Urology, University Hospital Heidelberg, Im Neuenheimer Feld 517, D-69120 Heidelberg, Germany; 2 Interdisciplinary Center for Bioinformatics, University of Leipzig, Härtelstrasse 16-18, D-04107 Leipzig, Germany; 3 Division of Biostatistics, German Cancer Research Center (DKFZ), Im Neuenheimer Feld 280, D-69120 Heidelberg, Germany; 4 Imaging Division, University Medical Center Utrecht, Heidelberglaan 100, 3584 CX Utrecht, The Netherlands; 5 Clinical Cooperation Unit Nuclear Medicine, German Cancer Research Center (DKFZ), Im Neuenheimer Feld 280, D-69120 Heidelberg, Germany; 6 Department of Radiology, German Cancer Research Center (DKFZ), Im Neuenheimer Feld 280, D-69120 Heidelberg, Germany; 7 Cancer Therapeutics Program and Department of Pathology, Hillman Cancer Center, University of Pittsburgh School of Medicine, 5117 Centre Avenue, Pittsburgh, PA 15213 USA; 8 Department of Medical Oncology, National Center for Tumor Diseases (NCT), University Hospital Heidelberg, Im Neuenheimer Feld 460, D-69120 Heidelberg, Germany; 9 Division of Medical Image Computing, German Cancer Research Center (DKFZ), Im Neuenheimer Feld 280, D-69120 Heidelberg, Germany; 10 Hamamatsu Tissue Imaging and Analysis Center (TIGA), BIOQUANT, University of Heidelberg, Im Neuenheimer Feld 267, D-69120 Heidelberg, Germany; 11 Functional Epigenomics, Center for Molecular Medicine Cologne (CMMC), University of Cologne, Robert-Koch-Strasse 21, D-50931 Cologne, Germany; 12 Pathology of the University Hospital Schleswig-Holstein, Campus Lübeck and the Research Center Borstel, Leibniz Lung Center, Ratzeburger Allee 160, D-23538 Lübeck and Parkallee 1-40, D-23845 Borstel, Germany; 13 Institute of Pathology, University Hospital Heidelberg, Im Neuenheimer Feld 224, D-69120 Heidelberg, Germany; 14 Department of Radiation Oncology, University Hospital Heidelberg, Im Neuenheimer Feld 400, D-69120 Heidelberg, Germany; 15 Department of Nuclear Medicine, University Hospital Heidelberg, Im Neuenheimer Feld 400, D-69120 Heidelberg, Germany; 16 Cancer Genome Research, German Cancer Research Center (DKFZ) and German Cancer Consortium (DKTK), Im Neuenheimer Feld 460, D-69120 Heidelberg, Germany; 17 Molecular Urooncology, University Hospital Heidelberg, Im Neuenheimer Feld 517, D-69120 Heidelberg, Germany; 18 Present Address: Department of Urology, University Hospital Nuremberg, Nuremberg, Germany; 19 Present Address: Institute of Pathology, University Hospital Mainz, Mainz, Germany; 20 Present Address: Pathology Rosenheim, Rosenheim, Germany; 21 Present Address: Department of Urology, University Hospital Essen, Essen, Germany

## Abstract

Magnetic resonance imaging (MRI) and prostate specific membrane antigen (PSMA)- positron emission tomography (PET)/computed tomography (CT)-imaging of prostate cancer (PCa) are emerging techniques to assess the presence of significant disease and tumor progression. It is not known, however, whether and to what extent lesions detected by these imaging techniques correlate with genomic features of PCa. The aim of this study was therefore to define a genomic index lesion based on chromosomal copy number alterations (CNAs) as marker for tumor aggressiveness in prostate biopsies in direct correlation to multiparametric (mp) MRI and ^68^Ga-PSMA-PET/CT imaging features. CNA profiles of 46 biopsies from five consecutive patients with clinically high-risk PCa were obtained from radiologically suspicious and unsuspicious areas. All patients underwent mpMRI, MRI/TRUS-fusion biopsy, ^68^Ga-PSMA-PET/CT and a radical prostatectomy. CNAs were directly correlated to imaging features and radiogenomic analyses were performed. Highly significant CNAs (≥10 Mbp) were found in 22 of 46 biopsies. Chromosome 8p, 13q and 5q losses were the most common findings. There was an strong correspondence between the radiologic and the genomic index lesions. The radiogenomic analyses suggest the feasibility of developing radiologic signatures that can distinguish between genomically more or less aggressive lesions. In conclusion, imaging features of mpMRI and ^68^Ga-PSMA-PET/CT can guide to the genomically most aggressive lesion of a PCa. Radiogenomics may help to better differentiate between indolent and aggressive PCa in the future.

## Introduction

Prostate cancer (PCa) is a leading cause of cancer-associated morbidity and mortality in men^1^. It is characterized by a high degree of inter- as well as intratumoral heterogeneity that is reflected by the clinical course, which ranges from indolent to rapidly progressing, lethal disease^2^. Markers to substratify patients according to their risk to develop disease recurrence at the time of diagnosis are largely missing. However, there is compelling evidence that the extent of genomic alterations, in particular chromosomal copy number alterations (CNAs), can convey prognostic information that is, at least in part, independent of the Gleason score^3–5^.

In recent years, considerable improvement has been made in the radiological detection of PCa. Multiparametric magnetic resonance tomography (mpMRI) has a sensitivity of 93% and a negative predictive value (NPV) of 89% for detecting significant PCa^6^ and has therefore become an integral part of the diagnosis of prostate cancer in many clinical centers. At the same time, the use of ^68^Ga-prostate specific membrane antigen (PSMA) positron emission tomography (PET)/computer tomography (CT) becomes more and more relevant for the staging of primary and recurrent prostate cancer^7–10^.

Despite the use of these emerging imaging techniques for prostate cancer diagnosis and staging, only a few attempts have been made to define the genomic correlates of imaging features^11,12^. Moreover, a broader, radiogenomics-based approach may help to further refine current imaging approaches with the ultimate goal to extract prognostic information from imaging data^13,14^.

In the present proof-of-concept study, we determined CNA profiles in biopsies from prostate cancer patients in direct correlation to mpMRI, PSMA-PET/CT and histopathology of the prostate. Our results show that the genomically most aggressive lesion of a tumor found in the MRI/TRUS-fusion biopsies (referred to as genomic index lesion) correlates with imaging features. These results provide a conceptual framework to further refine mpMRI- and PSMA-PET-based prostate cancer diagnosis and potentially prognosis.

## Material and Methods

### Patients and study design

Five consecutive patients with clinically high-risk prostate cancer were prospectively enrolled in this study. Written informed consent was obtained from each patient and for each procedure (mpMRI, MRI/TRUS-fusion biopsy, ^68^Ga-PSMA-11-PET/CT, radical prostatectomy). All five patients underwent mpMRI, followed by MRI/TRUS-fusion biopsy and, after conformation of prostate cancer, a ^68^Ga-PSMA-11-PET/CT. Repeat biopsies for genomic analyses were obtained from radiologically suspicious and unsuspicious areas at defined sites following diagnostic biopsies and tissue was snap frozen in liquid nitrogen. All five patients received a radical prostatectomy as part of a multimodal treatment strategy. All experimental protocols were approved by Ethics Committee of the Medical Faculty Heidelberg (vote S-085/2012). All patient-derived tumor specimens were collected under approval by the Ethics Committee of the Medical Faculty Heidelberg of the University of Heidelberg (votes 206/2005, 207/2005) after written informed consent was obtained from all patients. Tissue samples from the tissue bank of the NCT Heidelberg were provided in accordance with the regulations of the tissue bank and the approval of the Ethics Committee of the University of Heidelberg. All methods were carried out in accordance with relevant guidelines and regulations. Patient characteristics are shown in Table 1.

**Table 1:** Patient characteristics.

Patient	1	2	3	4	5
Age (yrs)	72	66	69	68	68
PSA (ng/ml)	79.5	35.7	28	19.7	26.6
**Stage**					
pT	T3a	T3b	T3b	T3a	T3b
pN	N0	N1	N1	N1	N1
cM	M0	M0	M0	M0	M0
Gleason score (Biopsy)	4 + 5	4 + 4	4 + 4	4 + 5	5 + 4
Number of suspect mpMRI lesions	1	2	1	3	2
Number of biopsies for genomic analysis	6	9	9	12	9
Number of biopsies with highly significant CNAs	4	4	4	3	7
Average number of highly significant CNAs in positive biopsies	4.25	6.25	4.5	3.7	2.1
Number of genomic index lesions	1	2	2	1	1
Number of highly significant CNAs in genomic index lesion(s)	7	7	6	6	4
Intra-prostatic location of genomic index lesion(s) (PI-RADS [v1])	Left midgland, posterior, lateral peripheral/transition zone (9p/10p)	Right midgland, posterior, midlobar peripheral zone (3p)	Right midgland, posterior, midlobar transition zone (3p)	Right midgland, posterior, midlobar transition zone (3p)	Left midgland, posterior, lateral transition zone (10p)
Gleason score at genomic index lesion(s)	4 + 5	4 + 4	4 + 4/4 + 3	4 + 4	Histopathologically no tumor
Imaging findings at genomic index lesion(s):					
Overall PI-RADS Score [v1]	5	5	5	5	5
T2	4	5	5	3	5
DWI	5	5	5	5	5
DCE	+	+	+	+	+
ADC mean	509	788	485	844	844
^68^Ga-PSMA-11-PET SUVmax	65.8	20.9	37.2	NA	34.7

### mpMRI and MRI/TRUS-fusion biopsy

All patients underwent mpMRI at a 3T system without an endorectal coil (Magnetom; Siemens, Erlangen, Germany) using the sequence parameters described in Suppl. Table 1. Images were analyzed by or under the supervision of expert uroradiologists in accordance with the 2012 European Society of Urogenital Radiology guidelines^15^ and, reflecting clinical routine at the time of image acquisition, reported according to PI-RADS (v1)^16^. Patients than underwent MRT/TRUS-fusion biopsy using the BiopSeesystem (MEDCOM, Darmstadt, Germany)^17^. Transperineal grid-directed targeted and systematic biopsy was performed in each man as described earlier^18^. In addition to the diagnostic biopsies taken for histopathological evaluation, between 6–13 repeat biopsies were taken from radiologically suspicious and unsuspicious areas using the same grid hole and depth of penetration as for the diagnostic biopsy.

### ^68^Ga-PSMA-11-PET/CT

After histopathological confirmation of prostate cancer, all patients underwent ^68^Ga-PSMA-11-PET/CT for further staging. One hour (±10 min) after intravenous injection of ^68^Ga-PSMA-11, synthesized as previously described^19^, scans were performed on a Biograph 6 PET/CT Scanner (Siemens, Erlangen, Germany). Initially, a CT scan (130 keV, 80 mAs; CareDose) without contrast medium was acquired. Corrected for dead time, scatter and decay, statistic emission scans were performed from the vertex to the proximal leg, requiring eight bed positions with 3 min per bed position. Image reconstruction was performed using CT-based attenuation correction with the ordered subset expectation maximization algorithm including four iterations with eight subsets and Gaussian filtering to an in-plane spatial resolution of 5 mm at full-width at half-maximum. Circular regions of interest were drawn in transaxial slices into areas with increased uptake and automatically adapted to a three-dimensional volume of interest with e.soft software (Siemens, Erlangen, Germany) at a 50% isocontour to calculate standardized uptake values (SUVs).

### Genome-wide methylation analysis and CNA profiles

A total of 46 biopsy cores were analyzed using Illumina’s Infinium Human Methylation 450 K BeadChip array. CNAs were inferred using the circular binary segmentation approach according to Feber *et al*.^20^. Spot intensities were normalized using one or more biopsies from non-cancerous areas for each individual tumor as reference. Highly significant CNAs were defined as log_2_R ratio <−0.2 or >0.2 and ≥10 Mbp in length. Biopsy sites with the maximum number of highly significant CNAs were defined as genomic index lesion. Pairwise correlation analysis was done using SOM containing 400 metagenes^21^.

### Image postprocessing and radiomic feature extraction

T2-weighted (T2w), DWI, DCE and ADC-map as well as PSMA-PET images were subjected to radiomic feature extraction. DCE parameter maps of Ktrans (volume transfer coefficient reflecting vascular permeability), Kep (flux rate constant), ve (extracellular volume fraction) and vp (plasma volume) were calculated using the extended Tofts model. ADC value maps (ADC1500) were calculated from b = 0 and b = 1500 s/mm^2^ (B1500) maps. In all imaging sequences the position where each single biopsy core was taken was marked as volume of interest using the medical imaging toolkit (MITK, www.mitk.org)^22^. Within these three-dimensional volumes of interests, 21 first-order features and 315 texture features (15 neighbourhood gray level difference, 90 run length, 168 gray level co-occurrence matrix based and 42 size zone features) were calculated for each of T2w, ADC1500, B1500, Ktrans, Kep, ve and vp maps. We did not include PET data as no data was available for patient 4 due to dynamic range suppression related to strong tracer activity in the urinary bladder. Together a total of 2352 radiomic features were calculated for each volume of interest. Of these we removed 442 parameters that were indeterminate due to division by zero or that had a variance of zero between observations. The remaining 1910 parameters were included into further analysis. First-order features depict the intensity of normalized imaging sequences using voxel intensity values to perform first-order statistics like means, standard deviation, kurtosis, skewness, uniformity, energy and entropy. Textural features reflect information about fine structural pattern in the gray-levels within the image. A detailed definition of radiomic features is given in Suppl. Table S2.

### Statistical analysis

After defining the most aggressive biopsy core(s) in each prostate using the amount of highly significant CNAs per core (genomic index lesion), all cores were marked on the biopsy template and localized on the final histopathology represented as virtual whole mount specimen by visual comparison. Both, the biopsy templates with index lesions and the virtual whole mounts were used by the radiologist to visually identify the location of the genomic index lesions on the images (mpMRI and ^68^Ga-PSMA-11-PET/CT). Explorative clustering analysis of radiomic features to several descriptors of aggressiveness (hsMut: number of highly significant CNAs, hiLes: presence of high-aggressiveness pattern, defined as genomic index lesion, MolTu: molecular tumor signature, defined as any presence of highly significant CNA) was performed using R version 3.3.0 and 3.4.2 (R Foundation for Statistical Computing, Vienna, Austria)^23^, package pheatmap [Raivo Kolde (2015). pheatmap: Pretty Heatmaps. R package version 1.0.8. https://CRAN.R-project.org/package=pheatmap].

## Results

### Identification and characterization of genomic index lesions in primary prostate cancer

Based on the work by Taylor *et al*.^3^, the number of CNAs was used to define prostate cancer aggressiveness. CNAs were inferred from genome-wide methylation data (Suppl. Fig. S1).

**Figure 1:**
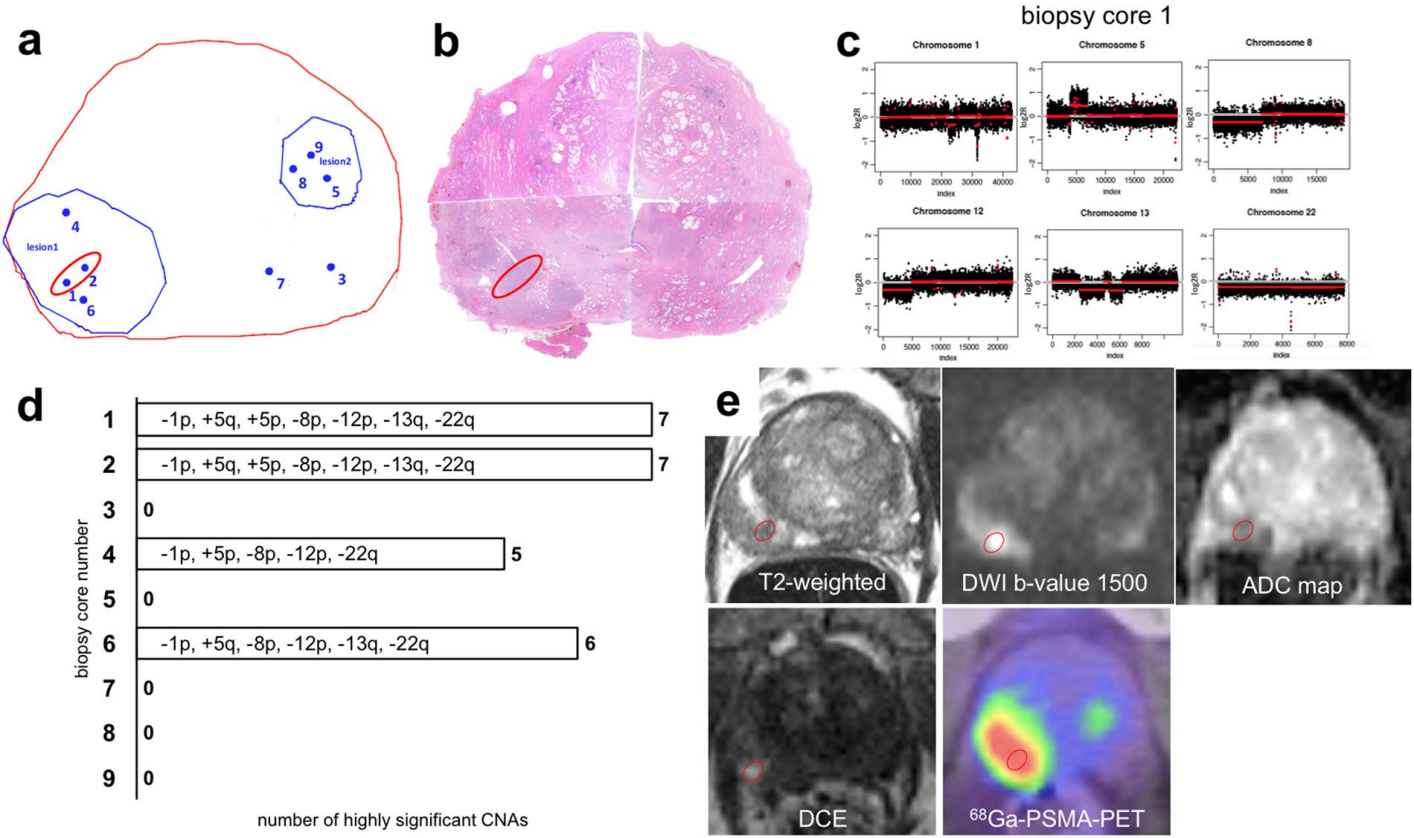
Correlation between genomic index lesions and imaging parameters in a representative patient. Synopsis of (**a**) projection map of MRI/TRUS fusion biopsy of patient 2. The prostate margin is shown in red, pre-biopsy mpMRI suspicious lesions in blue and individual biopsy cores that were sent for genomic testing as blue dots. Genomic index lesions are encircled in red; (**b**) virtual whole mount of the prostatectomy specimen with the red circle matching the genomic index lesion by visual comparison; (**c**) CNA profiles of the genomic index lesion; (**d**) overview of CNAs of all biopsies harboring highly significant alterations; (**e**) mpMRI components (T2w, DWI b-value = 1500 s/mm^2^, ADC map and early arterial phase of DCE-MRI) and ^68^Ga-PSMA-11-PET/CT are shown. The visually matched location of the genomic index lesion on individual components is superimposed on the axial slices centered on the mpMRI lesion used during MRI/TRUS biopsy.

A total of 86 highly significant (i.e, ≥10 Mbp in length) CNAs were identified in 22 of the 46 biopsies obtained from the five prostates. Losses of chromosome 8p were the most common finding and observed in 14 cores and four patients. The second most frequent CNAs were 13q deletions found in 13 cores and four patients followed by 5q losses found in nine biopsy cores from three patients. Further recurrent chromosomal deletions were found for 1p, 2q, 6p, 6q, 11q, 12p, 15q and 22q. Recurrent chromosomal gains were less common and found for 3q, 5p, 5q and 8q (Fig. 1; Suppl. Figs S2–4).

A pairwise correlation analysis of all 400 SOM metagenes using the genome-wide DNA methylation array data confirmed a correlation between methylation status and the presence of highly significant CNAs. There was a patient-wise clustering as well as a clear separation between biopsies harboring highly significant CNAs and those that did not (Suppl. Fig. S1).

Genomic index lesions were identified in all men and consisted of a single biopsy, or, in two patients, two biopsy cores (Patient 2 and 3) that contained the highest number of highly significant CNAs found among all biopsies. The median number of highly significant CNAs in the seven genomic index lesion identified was six (range, four to seven).

We compared the extent of overlap between the genomic index lesions and the radiologic index lesion. One patient had to be excluded from this analysis because of samples for genomic analyses were not taken from the radiologic index lesion (Patient 1; data not shown). In all of the other four patients, the genomic index lesion correlated with radiologically highly suspicious lesions (Patients 2, 3,4 and 5; Fig. 1; Figs S2–S4; see also below). In four of the five patients analyzed, the location of the genomic index lesion also correlated with the highest Gleason pattern found in the diagnostic biopsy (Table 1).

### Correlation of the genomic index lesions with imaging features

Clinical pre-biopsy radiological assessment had identified highly suspicious lesions PI-RADS (v1) score of 5 on mpMRI in each of the five patients. These PI-RADS lesions were used for stereotactic targeting during the MRI/TRUS fusion biopsy procedure. Biopsy locations and targeted lesions are shown schematically in Fig. 1a and Suppl. Figs S2–4. The prostate is outlined in red and the mpMRI index lesions in blue. Biopsy cores selected for histopathologic and genomic analyses are numbered and shown as blue dots. The genomic index lesion(s) is/are indicated by a red circle. Matching of the genomic index lesion to imaging features is shown in Fig. 1e and Suppl. Figs S2E–4E.

In all four fully evaluable patients, the genomic index lesion clearly localized to a highly suspicious area on mpMRI. PET imaging showed diffuse tracer activity in patient 5 without clear focality, and could not be evaluated within the prostate in patient 4 due to dynamic range suppression by high bladder activity. In patients 2 and 3, a clear localization of the genomic index lesion into regions of highly abnormal tracer uptake was detected. Thus, suspect mpMRI, ^68^Ga-PSMA-11-PET/CT and genetic index lesions showed an excellent overlap in all four of four patients included in this analysis.

**Figure 2:**
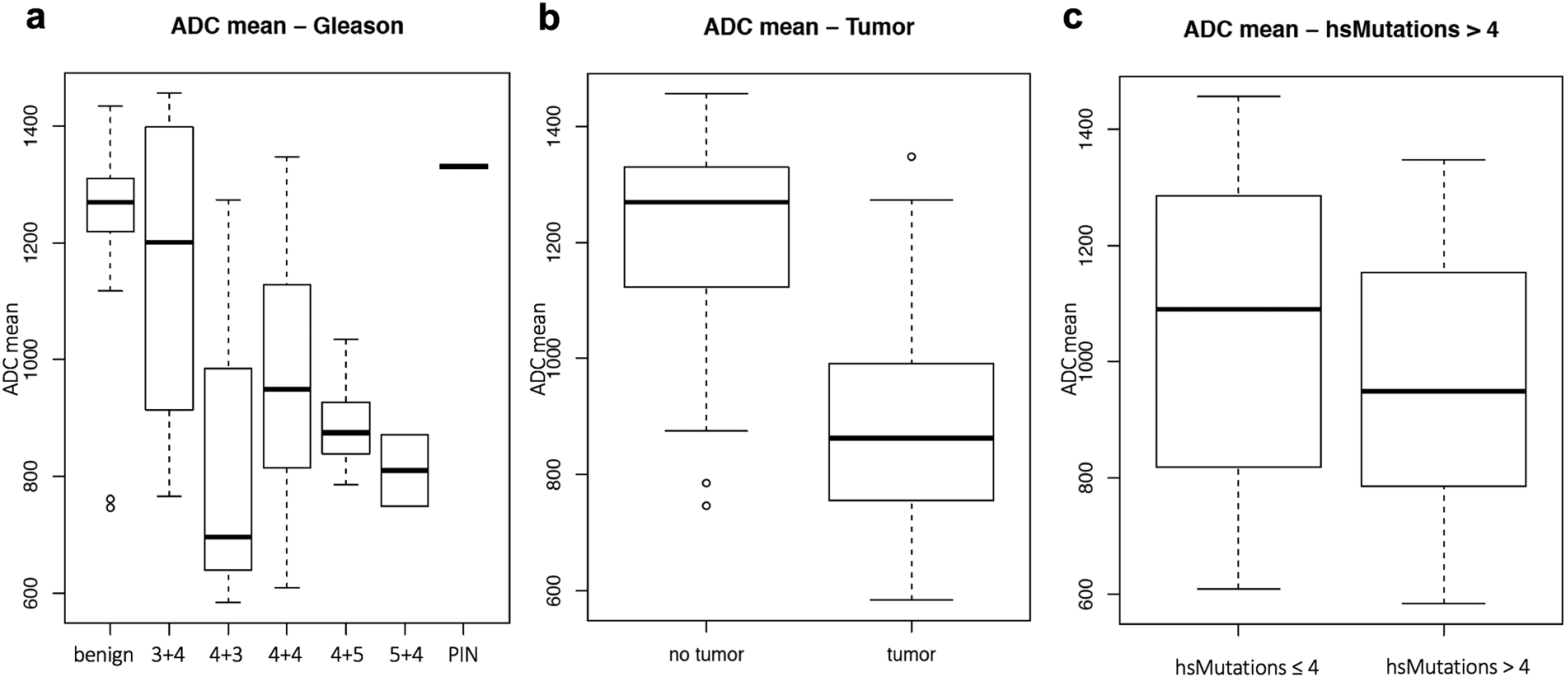
Association between ADC and important markers of tumor aggressiveness. Association between mean apparent diffusion (ADC) values and (**a**) genetic tumor signature, (**b**) Gleason score, (**c**) highly significant mutations >4.

When comparing the mean ADC values of biopsy cores with or without a genetic tumor signature defined as ≥one highly significant CNA, of biopsy cores grouped according to Gleason Score or of biopsy cores with more or less than 4 highly significant mutations we found an association between lower mean ADC values and increasing tumor aggressiveness (Fig. 2).

### Radiogenomic analysis

**Figure 3:**
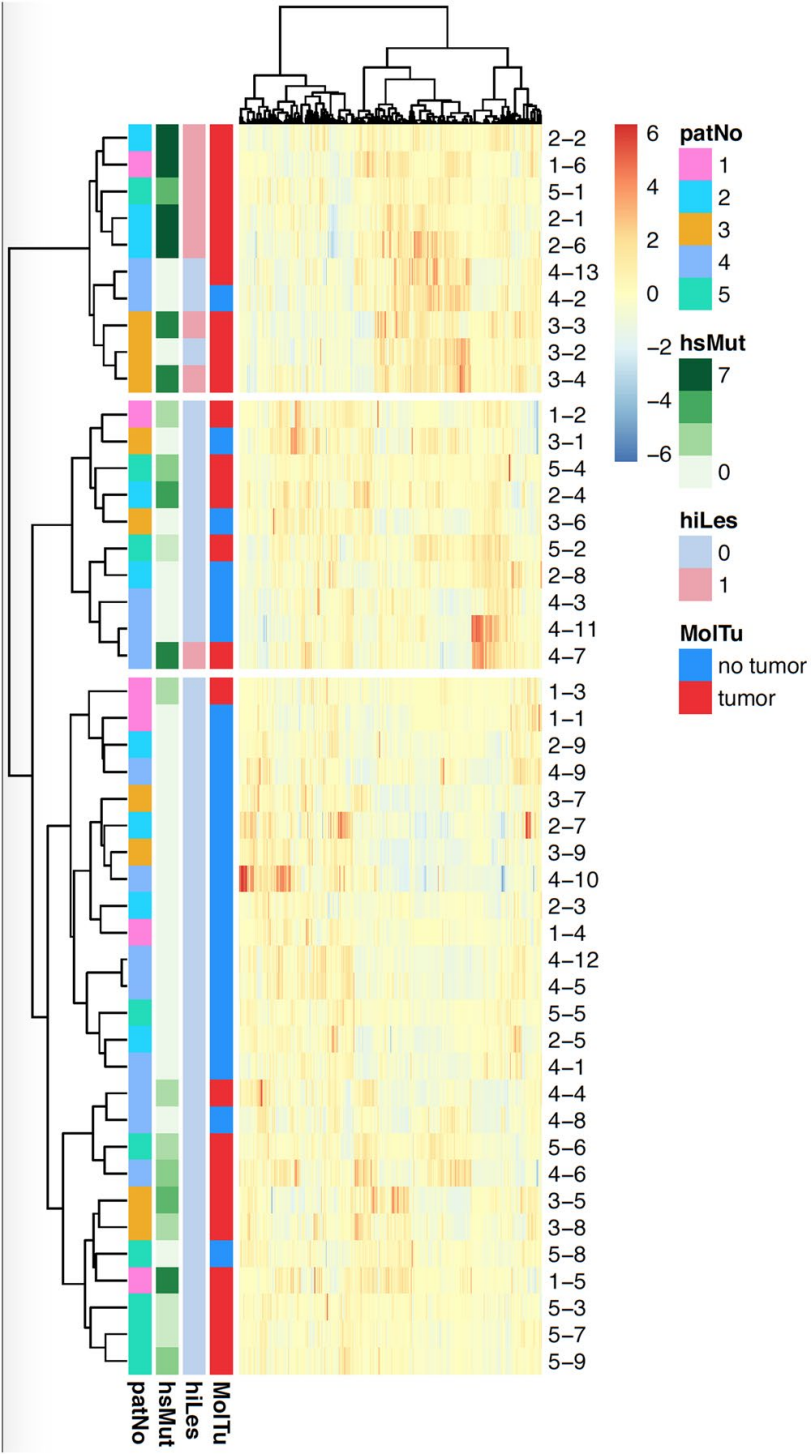
Patient-corrected clustering analysis. Patient-corrected clustering analysis of radiomic features using Ward’s D linkage and correlation distance for all radiomic features (patNo = Patient number, hsMut = number of highly significant copy number alterations [CNAs], hiLes = presence of high-aggressiveness pattern, defined as genomic index lesion, MolTu = molecular tumor signature).

Patient-corrected (patients were considered as batches i.e., a patient-specific intercept was removed) clustering analysis of standardized radiomic features using Ward’s D linkage and correlation distance was performed for all radiomic features (Fig. 3). Clustering using all radiomics features identified nearly all but one highly aggressive biopsy cores harbouring the genomic index lesion in one cluster (top cluster, light pink for hiLes) and also demonstrated a much higher frequency of tumor signatures compared to the other two clusters (red MolTu signature). The same pattern is also found in terms of a higher frequency of a larger number of highly significant mutations in this cluster (dark green tones in hsMut column). *Vice versa*, most of the non-tumor bearing cores were clustered in one cluster as well (bottom cluster, blue MolTU signature) which did also not feature any genomic index lesions (light blue in hiLes column). Thus, clustering demonstrates how radiogenomic analyses may help to identify the most aggressive area within the prostate.

## Discussion

The present study is among the first to correlate imaging features of prostate cancer with genomic data using the number of CNAs as “gold standard” to define tumor aggressiveness^3^. Application of this concept to biopsy cores allowed us to identify genomic index lesions i.e, lesions with the highest number of larger CNAs, for each tumor analyzed. We found a strong correlation between the latter and mpMRI and ^68^Ga-PSMA-11-PET/CT features.

The ability to cluster cores with a high amount of CNAs using radiomic features is only explorative and limited by the small patient number in this proof-of-concept study. However, we believe that radiomic feature extraction is a promising approach and demonstrate the feasibility of developing radiologic signatures that distinguish areas according to the number of CNAs.

A high CNA burden has been associated with higher biochemical recurrence rate, metastasis and early cancer-specific mortality that was to some extend independent of the Gleason score^3,24–27^. In the present study, the overall Gleason score and the Gleason score at the genomic index lesion showed a strong correlation with the exception of one patient (Patient 4), where a Gleason pattern 5 component was not found.

We identified several foci with different CNA alterations within each prostate, which is in line with the known genomic intratumoral heterogeneity of prostate cancer^12,28–30^. Our results therefore also highlight the fact that random biopsy sampling of the prostate bears the risk of missing the most important i.e., genomically most aggressive, tumor clone.

MpMRI and ^68^Ga-PSMA-11-PET/CT have become promising parts of prostate cancer diagnosis and clinical decision-making^6–9^. Hence, imaging signatures as surrogate for genomic outcome variables could help to provide more advanced risk stratification and also to predict treatment responses. However, only a few studies have made an attempt to explore radiogenomics as a patient stratification tool^11,12,14,31,32^. Using the Decipher® genomic classifier and a genomic Gleason grade classifier, our group recently demonstrated an excellent correlation between MRI targeted biopsy genomics and radical prostatectomy genomics and a strong association between a high Decipher® or Gleason grade classifier score and a high PI-RADS score^12^. In a similar approach, Stoyanova and colleagues correlated imaging features from mpMRI suspicious and normal appearing prostate regions with commercially available genomic classifiers^11^. They found a significant correlation between quantitative imaging features and gene expression changes associated with an adverse patient outcome^11^. However, a number of other studies showed more heterogenous results when correlating imaging features to genomic alterations^14,31,32^.

Our study has several limitations. First, the sample size is too small for more detailed statistical analyses. However, this study was designed to test the feasibility of our hypothesis and provide the framework for larger prospective studies. Second, the registration of the core position on mpMRI and ^68^Ga-PSMA-11-PET/CT was done only in a semi-automatic fashion and smaller registration errors can therefore not be fully excluded. Third we only evaluated high-risk patients and fresh tumor sampling from radical prostatectomy specimens was not performed.

In conclusion, our study represents the first approach combining mpMRI, ^68^Ga-PSMA-11-PET/CT, radiomics and genome-wide CNA analyses in prostate cancer diagnosis and risk stratification. It demonstrates that imaging features of mpMRI and ^68^Ga-PSMA-11-PET/CT can guide to the genomically most aggressive region within the prostate. Radiogenomic analyses may further help to better differentiate between indolent and aggressive cancer in the future.

## Electronic supplementary material

Supplementary Information

## Data Availability

The data set generated during and/or analysed during the current study are available from the corresponding authors on reasonable request.

## References

[1] Siegel RL, Miller KD, Jemal A (2018). Cancer statistics, 2018. CA. Cancer J. Clin..

[2] Spratt DE, Zumsteg ZS, Feng FY, Tomlins SA (2016). Translational and clinical implications of the genetic landscape of prostate cancer. Nat. Rev. Clin. Oncol..

[3] Taylor BS (2010). Integrative Genomic Profiling of Human Prostate Cancer. Cancer Cell.

[4] Lapointe J (2007). Genomic Profiling Reveals Alternative Genetic Pathways of Prostate Tumorigenesis. Cancer Res..

[5] Williams JL, Greer PA, Squire JA (2014). Recurrent copy number alterations in prostate cancer: an in silico meta-analysis of publicly available genomic data. Cancer Genet..

[6] Ahmed HU (2017). Diagnostic accuracy of multi-parametric MRI and TRUS biopsy in prostate cancer (PROMIS): a paired validating confirmatory study. The Lancet.

[7] Rhee H (2016). Prostate Specific Membrane Antigen Positron Emission Tomography May Improve the Diagnostic Accuracy of Multiparametric Magnetic Resonance Imaging in Localized Prostate Cancer. J. Urol..

[8] Fendler W. P., Schmidt D. F., Wenter V., Thierfelder K. M., Zach C., Stief C., Bartenstein P., Kirchner T., Gildehaus F. J., Gratzke C., Faber C. (2016). 68Ga-PSMA PET/CT Detects the Location and Extent of Primary Prostate Cancer. Journal of Nuclear Medicine.

[9] Rahbar K (2016). Correlation of Intraprostatic Tumor Extent with 68Ga-PSMA Distribution in Patients with Prostate Cancer. J. Nucl. Med..

[10] Afshar-Oromieh A (2013). PET imaging with a [68Ga]gallium-labelled PSMA ligand for the diagnosis of prostate cancer: biodistribution in humans and first evaluation of tumour lesions. Eur. J. Nucl. Med. Mol. Imaging.

[11] Stoyanova R (2016). Association of multiparametric MRI quantitative imaging features with prostate cancer gene expression in MRI-targeted prostate biopsies. Oncotarget.

[12] Radtke, J. P. *et al*. Transcriptome Wide Analysis of Magnetic Resonance Imaging-targeted Biopsy and Matching Surgical Specimens from High-risk Prostate Cancer Patients Treated with Radical Prostatectomy: The Target Must Be Hit. *Eur*. *Urol*. *Focus*10.1016/j.euf.2017.01.005. 10.1016/j.euf.2017.01.005 28753844

[13] Lambin P (2012). Radiomics: Extracting more information from medical images using advanced feature analysis. Eur. J. Cancer.

[14] Jamshidi N (2017). Multiregional Radiogenomic Assessment of Prostate Microenvironments with Multiparametric MR Imaging and DNA Whole-Exome Sequencing of Prostate Glands with Adenocarcinoma. Radiology.

[15] Barentsz JO (2012). ESUR prostate MR guidelines 2012. Eur. Radiol..

[16] Hamoen EHJ, de Rooij M, Witjes JA, Barentsz JO, Rovers MM (2015). Use of the Prostate Imaging Reporting and Data System (PI-RADS) for Prostate Cancer Detection with Multiparametric Magnetic Resonance Imaging: A Diagnostic Meta-analysis. Eur. Urol..

[17] Hadaschik BA (2011). A Novel Stereotactic Prostate Biopsy System Integrating Pre-Interventional Magnetic Resonance Imaging and Live Ultrasound Fusion. J. Urol..

[18] Radtke JP (2015). Comparative Analysis of Transperineal Template Saturation Prostate Biopsy Versus Magnetic Resonance Imaging Targeted Biopsy with Magnetic Resonance Imaging-Ultrasound Fusion Guidance. J. Urol..

[19] Eder M (2012). 68Ga-Complex Lipophilicity and the Targeting Property of a Urea-Based PSMA Inhibitor for PET Imaging. Bioconjug. Chem..

[20] Feber A (2014). Using high-density DNA methylation arrays to profile copy number alterations. Genome Biol..

[21] Löffler-Wirth H, Kalcher M, Binder H (2015). oposSOM: R-package for high-dimensional portraying of genome-wide expression landscapes on bioconductor. Bioinforma. Oxf. Engl..

[22] Nolden M (2013). The Medical Imaging Interaction Toolkit: challenges and advances. Int. J. Comput. Assist. Radiol. Surg..

[23] R Core Team. R: A Langugage and Environment for Statistical Computing. Vienna, Austria: R Foundation for Statistical Computing. (2014).

[24] Hieronymus H (2014). Copy number alteration burden predicts prostate cancer relapse. Proc. Natl. Acad. Sci. USA.

[25] Lalonde E (2014). Tumour genomic and microenvironmental heterogeneity for integrated prediction of 5-year biochemical recurrence of prostate cancer: a retrospective cohort study. Lancet Oncol..

[26] Liu Wennuan, Xie Chunmei C., Thomas Christopher Y., Kim Seong-Tae, Lindberg Johan, Egevad Lars, Wang Zhong, Zhang Zheng, Sun Jishan, Sun Jielin, Koty Patrick P., Kader A. Karim, Cramer Scott D., Bova G. Steven, Zheng S. Lilly, Grönberg Henrik, Isaacs William B., Xu Jianfeng (2013). Genetic markers associated with early cancer-specific mortality following prostatectomy. Cancer.

[27] Abeshouse A (2015). The Molecular Taxonomy of Primary Prostate. Cancer. Cell.

[28] Cooper CS (2015). Analysis of the genetic phylogeny of multifocal prostate cancer identifies multiple independent clonal expansions in neoplastic and morphologically normal prostate tissue. Nat. Genet..

[29] Boutros PC (2015). Spatial genomic heterogeneity within localized, multifocal prostate cancer. Nat. Genet..

[30] Gundem G (2015). The evolutionary history of lethal metastatic prostate cancer. Nature.

[31] Renard-Penna R (2015). Multiparametric Magnetic Resonance Imaging Predicts Postoperative Pathology but Misses Aggressive Prostate Cancers as Assessed by Cell Cycle Progression Score. J. Urol..

[32] McCann SM (2016). Quantitative Multiparametric MRI Features and PTEN Expression of Peripheral ZoneProstate Cancer: A Pilot Study. Am. J. Roentgenol..

