# Supplementary material for: Correlation between genomic index lesions and mpMRI and 68Ga-PSMA-PET/CT imaging features in primary prostate cancer
Source: Sci Rep. 2018 Nov 12;8:16708. doi: 10.1038/s41598-018-35058-3 (PMC6232089; doi:10.1038/s41598-018-35058-3)
Supplement: Supplementary file 1 — Supplementary Information [file 41598_2018_35058_MOESM1_ESM.docx]

**SUPPLEMENTARY INFORMATION**

**Correlation between genomic index lesions and mpMRI and ^68^Ga-PSMA-PET/CT imaging features in primary prostate cancer**

Claudia Kesch^1^, Jan-Philipp Radtke^1^, Axel Wintsche^2^, Manuel Wiesenfarth^3^, Mariska Luttje^4^, Claudia Gasch^1^, Svenja Dieffenbacher^1^, Carine Pecqueux^1^, Dogu Teber^1^, Gencay Hatiboglu^1^, Joanne Nyarangi-Dix^1^, Tobias Simpfendörfer^1^, Gita Schönberg^1^, Antonia Dimitrakopoulou-Strauss^5^, Martin Freitag^6^, Anette Duensing^7^, Carsten Grüllich^8^, Dirk Jäger^8^, Michael Götz^9^, Niels Grabe^10^, Michal-Ruth Schweiger^11^, Sascha Pahernik^1+^, Sven Perner^12^, Esther Herpel^13^, Wilfried Roth^13++^, Kathrin Wieczorek^13+++^, Klaus Maier-Hein^9^, Jürgen Debus^14^, Uwe Haberkorn^5, 15^, Frederik Giesel^5, 15^, Jörg Galle^2^, Boris Hadaschik^1++++^, Heinz-Peter Schlemmer^6*^, Markus Hohenfellner^1*^, David Bonekamp^6*^, Holger Sültmann^16*^, and Stefan Duensing^1,17*^

*These authors jointly supervised the study.

1 Department of Urology, University Hospital Heidelberg, Im Neuenheimer Feld 517, D-69120 Heidelberg, Germany

2 Interdisciplinary Center for Bioinformatics, University of Leipzig, Härtelstrasse 16-18, D-04107 Leipzig, Germany

3 Division of Biostatistics, German Cancer Research Center (DKFZ), Im Neuenheimer Feld 280, D-69120 Heidelberg, Germany

4 Imaging Division, University Medical Center Utrecht, Heidelberglaan 100, 3584 CX Utrecht, The Netherlands

5 Clinical Cooperation Unit Nuclear Medicine, German Cancer Research Center (DKFZ), Im Neuenheimer Feld 280, D-69120 Heidelberg, Germany

6 Department of Radiology, German Cancer Research Center (DKFZ), Im Neuenheimer Feld 280, D-69120 Heidelberg, Germany

7 Cancer Therapeutics Program, Hillman Cancer Center, 5117 Centre Avenue, Pittsburgh, PA 15213, USA

8 Department of Medical Oncology, National Center for Tumor Diseases (NCT), University Hospital Heidelberg, Im Neuenheimer Feld 460, D-69120 Heidelberg, Germany

9 Division of Medical Image Computing, German Cancer Research Center (DKFZ), Im Neuenheimer Feld 280, D-69120 Heidelberg, Germany

10 Hamamatsu Tissue Imaging and Analysis Center (TIGA), BIOQUANT, University of Heidelberg, Im Neuenheimer Feld 267, D-69120 Heidelberg, Germany

11 Functional Epigenomics, Center for Molecular Medicine Cologne (CMMC), University of Cologne, Robert-Koch-Strasse 21, D-50931 Cologne, Germany

12 Pathology of the University Hospital Schleswig-Holstein, Campus Lübeck and the Research Center Borstel, Leibniz Lung Center, Ratzeburger Allee 160, D-23538 Lübeck and Parkallee 1-40, D-23845 Borstel, Germany

13 Institute of Pathology, University Hospital Heidelberg, Im Neuenheimer Feld 224, D-69120 Heidelberg, Germany

14 Department of Radiation Oncology, University Hospital Heidelberg, Im Neuenheimer Feld 400, D-69120 Heidelberg, Germany

15 Department of Nuclear Medicine, University Hospital Heidelberg, Im Neuenheimer Feld 400, D-69120 Heidelberg, Germany

16 Cancer Genome Research, German Cancer Research Center (DKFZ) and German Cancer Consortium (DKTK), Im Neuenheimer Feld 460, D-69120 Heidelberg, Germany

17 Molecular Urooncology, University Hospital Heidelberg, Im Neuenheimer Feld 517, D-69120 Heidelberg, Germany

Current address:

+ Department of Urology, University Hospital Nuremberg, Nuremberg, Germany

++ Institute of Pathology, University Hospital Mainz, Mainz, Germany

+++ Pathology Rosenheim, Rosenheim, Germany

++++ Department of Urology, University Hospital Essen, Essen, Germany

Correspondence should be addressed to:

Holger Sültmann, Cancer Genome Research, German Cancer Research Center (DKFZ), National Center for Tumor Diseases (NCT) Heidelberg, and German Consortium for Translational Cancer Research (DKTK), Im Neuenheimer Feld 460, D-69120 Heidelberg, Germany, Phone +49-6221-565934, Fax: +49-6221-565382, E-mail: h.sueltmann@dkfz-heidelberg.de OR

Stefan Duensing, Section of Molecular Urooncology, University Hospital Heidelberg, Medical Faculty Heidelberg, Im Neuenheimer Feld 517, 69120 Heidelberg, Germany, Tel: +49-6221-56-6255, Fax: +49-6221-56-7659, E-mail: stefan.duensing@med.uni-heidelberg.de

**Table S1. mpMRI sequence parameters**

| **Parameter** | **T1 TSE** | **T2 TSE** | **epi-2D** | **DCE Twist** |
| --- | --- | --- | --- | --- |
| **TR (ms) / TE (ms)** | 792 / 11 | 5120 / 143 | 3100 / 52 | 4.42 / 2.2 |
| **Flip angle (°)** | 90 | 90 | 90 | 15 |
| **ETL length / Epi-factor** | 72 | 12 | 96 | - |
| **Averages** | 2 | 4 | 5 | - |
| **b-value** | - | - | 0, 50, 100, 150, 100,250, 800, 1000 | - |
| **Section thickness (mm)** | 5 | 3 | 3 | 1.5 |
| **FOV (mm)** | 320 | 300 | 280 | 400 |
| **Resolution** | 1.1 x 1.0 | 0.8 x 0.7 | 2.2 x 2.2 | 1.6 x 1.6 |
| **Aquisition time (min)** | 03:51 | 04:14 | 05:04 | 05:18 |
| TR – Repetition Time; TE – Echo Time; ETL – Echo Train Length; FOV – Field of View; epi – Echo Planar Imaging; TSE – Turbo Spin Echo; TWIST – Time resolved angiography With Interleaved Stochastic Trajectories; SE – Spin Echo; DCE - Dynamic Contrast Enhancement | | | | |

**Table S2****. Radiomic features**

Within each VOI, first-order features (FO) and texture features (TF) were calculated. FO represent the voxel intensity distribution by first-order statistics, including mean, standard deviation, kurtosis, skewness, uniformity, energy and entropy. TF were based on co-occurrence (1, 2, 3), run-length (64, 128, 256), size zone (64, 128, 256) and neighborhood gray level difference (64,128,256) based features. Co-occurrence features were calculated on the basis of gray level co-occurrence matrices (GLCM) which asses the combination of neighboring intensities, while run-length based features represent measures the presence of line-like structures by using a gray level run length matrix (GLRLM). Neighborhood gray level dependence features capture the coarseness of small neighborhoods by assessing the number of similar intensities. Gray Level Size Zone (GLSZ) textures represent the distribution of the amount and size of connected intensities of the same value. Important definitions of features are provided below.

**Group 1: First order statistics**

First order statistics are descriptive of the distribution of gray values within an image. Let $\boldsymbol{X}$ and $\boldsymbol{X}_{\boldsymbol{all}}$ denote the intensity of all voxels within a Region of Interest (ROI) with $N$ voxels and the whole image, respectively. Then, the gray value mean value within the ROI is $\overline{X}$**.**

$\boldsymbol{P}$ then denotes the probability vector of the first order histogram with $N_{l}$ discrete bins. $\boldsymbol{B}$ denotes the center gray values.

1. **Covered Image intensity Rage**:

$$intensity range:= \frac{\max\boldsymbol{X}-\min\boldsymbol{X}}{\max\boldsymbol{X}_{\boldsymbol{all}}-\min\boldsymbol{X}_{\boldsymbol{all}}}$$

2. **Energy**:

$$energy := \sum_{i}^{N_{l}} \left( N*\boldsymbol{P}\left( i \right) \right)^{2}$$

3. **Entropy**:

$$entropy := \sum_{i}^{N_{l}} \boldsymbol{P}\left( i \right)*\log_{2} \boldsymbol{P}\left( i \right)$$

4. **Kurtosis:**

$$kurtosis := \frac{\sum_{i}^{N_{l}} \boldsymbol{P}\left( i \right)*\left( \boldsymbol{B}\left( i \right)- \overline{X} \right)^{4}}{\left( \sqrt{\sum_{i}^{N_{l}} \boldsymbol{P}\left( i \right)*\left( \boldsymbol{B}\left( i \right)-\overline{X} \right)} \right)^{4}}$$

5. **Maximum**:
   The maximum intensity value in $\boldsymbol{X}$, i.e. $\max\boldsymbol{X}$
6. **Mean**:

$$mean = \overline{X}:= \frac{1}{N}\sum_{i}^{N} X(i)$$

7. **Mean absolute deviation:**

$$mean absolute deviation := \sum_{i}^{N_{l}} \boldsymbol{P}\left( i \right)*(\boldsymbol{B}\left( i \right)- \overline{X})$$

8. **Median**:
   The median value of all intensity values in $\boldsymbol{X}$, i.e. the gray value $x_{Median}$ for which

$$\left| \left\{ x | x \in\boldsymbol{X}and x<x_{Median} \right\} \right|= \left| \left\{ x | x \in\boldsymbol{X}and x_{Median}<x \right\} \right|$$

9. **Minimum**:
   The minimum intensity value in $\boldsymbol{X}$, i.e. $\min\boldsymbol{X}$
10. **No. of Voxels:**The number of voxels in $\boldsymbol{X}$**,** i.e. $no. of voxels := \left| \boldsymbol{X} \right|$
11. **Range:**

$$range:= max \boldsymbol{X}-min \boldsymbol{X}$$

12. **Root Means Square (RMS)**:

$$RMS:= \sum_{i}^{N_{l}} \boldsymbol{P}\left( i \right)*\boldsymbol{B}\left( i \right)^{2}$$

13. **Skewness**:

$$kurtosis := \frac{\sum_{i}^{N_{l}} \boldsymbol{P}\left( i \right)*\left( \boldsymbol{B}\left( i \right)- \overline{X} \right)^{3}}{\left( \sqrt{\sum_{i}^{N_{l}} \boldsymbol{P}\left( i \right)*\left( \boldsymbol{B}\left( i \right)-\overline{X} \right)} \right)^{3}}$$

14. **Standard deviation**:

$$standard deviation := \sqrt{\frac{1}{N-1}\sum_{i}^{N} \left( \boldsymbol{X}\left( i \right)- \overline{X} \right)^{2}}$$

15. **Sum of intensities**:

$$sum of intensities:= \sum_{i}^{N} \boldsymbol{X}(i)$$

16. **Uniformity**:

$$uniformity := \sum_{i}^{N_{l}} \boldsymbol{P}\left( i \right)^{2}$$

17. **Variance**:

$$standard deviation := \frac{1}{N-1}\sum_{i}^{N} \left( \boldsymbol{X}\left( i \right)- \overline{X} \right)^{2}$$

**Group 3: Texture features**

The first order statistics in Group 1 do not distinguish the order and distribution of the gray value information within the ROI. The features in this group, Gray-Level-co-occurences or Run-length based features, describe the texture and distribution of gray values.

**Gray-level co-occurrence based texture features**

Gray-level co-occurrence matrix based texture features quantitatively describe the image texture in an ROI by examining co-occurrences of similar gray values in multiple directions at defined distances from the center voxel. Gray value information is discretized by binning into $N_{g}$bins. Then, a gray-level co-occurrence matrix (GLCM) $\boldsymbol{P}$ with the size $N_{g}\times N_{g}$ is calculated, as follows: The $\left( i,j \right)$th element of the matrix is defined as the number of times a voxels is binned into bin $i$ and the voxel in a distance $\delta$ and direction $\alpha$ is binned in bin $j$. Following is two-dimensional examples image I and the corresponding GLCM for $\delta=1, 2, 3$ and horizontal direction:

| I= | 2 | 4 | 1 | 1 | 2 |  | GLCM= | 1 | 2 | 2 | 0 | 0 |
| --- | --- | --- | --- | --- | --- | --- | --- | --- | --- | --- | --- | --- |
|  | 1 | 3 | 5 | 5 | 1 |  |  | 1 | 0 | 2 | 1 | 1 |
|  | 2 | 3 | 4 | 2 | 3 |  |  | 1 | 0 | 0 | 1 | 2 |
|  | 1 | 2 | 5 | 2 | 1 |  |  | 1 | 1 | 0 | 0 | 0 |
|  | 5 | 3 | 1 | 3 | 5 |  |  | 1 | 1 | 1 | 0 | 1 |

We calculated the GLCM-based features in 3D, reporting the mean and standard deviation of all features calculated on all 13 possible directions and a pixel distance of either$\delta=1, 2, 3$.

Definitions used during the definition of the features:

- $N_{g}:$ Number of discrete intensity levels in the image
- $\boldsymbol{P}_{\boldsymbol{\alpha,\delta}}\left( i,j \right)=\boldsymbol{P}(i,j)$: Probability of co-occurrence matrix for the pair $i,j$.
- $\mu$ be the mean of $\boldsymbol{P}(i,j)$
- $\sigma$ be the standard deviation of $\boldsymbol{P}(i,i)$
- $\boldsymbol{P}_{\boldsymbol{x}}\left( i \right)=\sum_{j}^{N_{g}} \boldsymbol{P}(i,j)$ : marginal row probabilities
- $\mu_{x}$ be mean of $\boldsymbol{P}_{\boldsymbol{x}}\left( i \right)$
- $\sigma_{x}$ be the standard deviation of $\boldsymbol{P}_{\boldsymbol{x}}\left( i \right)$
- $\boldsymbol{P}_{\boldsymbol{x+y}}\left( k \right):= \sum_{i}^{N_{g}} \sum_{j}^{N_{g}} P\left( i,j \right) , i+j=k$
- $\boldsymbol{P}_{\boldsymbol{x-y}}\left( k \right):= \sum_{i}^{N_{g}} \sum_{j}^{N_{g}} P\left( i,j \right) , | i-j |=k$

**18. – 23. Autocorrelation (mean and std.dev. for** $\boldsymbol{=(}\boldsymbol{1,2,3)}$ **)**

$$autocorrelation :=\sum_{i}^{N_{g}} \sum_{j}^{N_{g}} i*j*\boldsymbol{P}(i,j)$$

**24. – 29. Cluster Prominence (mean and std.dev. for** $\boldsymbol{=(}\boldsymbol{1,2,3)}$ **)**

$$cluster prominence :=\sum_{i}^{N_{g}} \sum_{j}^{N_{g}} \left( i+j-2\mu\right)^{4}\boldsymbol{P}\left( i,j \right)$$

**30. – 35. Cluster Shade** **(mean and std.dev. for** $\boldsymbol{=(}\boldsymbol{1,2,3)}$ **)**

$$cluster shade :=\sum_{i}^{N_{g}} \sum_{j}^{N_{g}} \left( i+j-2\mu\right)^{3}\boldsymbol{P}\left( i,j \right)$$

**36. – 41. Cluster Tendency (mean and std.dev. for** $\boldsymbol{=(}\boldsymbol{1,2,3)}$ **)**

$$cluster tendency :=\sum_{i}^{N_{g}} \sum_{j}^{N_{g}} \left( i+j-2\mu\right)^{2}\boldsymbol{P}\left( i,j \right)$$

**42. – 47. Contrast / Inertia (mean and std.dev. for** $\boldsymbol{=(}\boldsymbol{1,2,3)}$ **)**

$$contrast :=\sum_{i}^{N_{g}} \sum_{j}^{N_{g}} \left( i-j \right)^{2}\boldsymbol{P}(i,j)$$

**48. – 53. Correlation (mean and std.dev. for** $\boldsymbol{=(}\boldsymbol{1,2,3)}$ **)**

$$correlation:=\frac{1}{\sigma}\sum_{i}^{N_{g}} \sum_{j}^{N_{g}} \left( i-\mu\right)\left( j-\mu\right)\boldsymbol{P}(i,j)$$

**54. – 59. Difference Average (mean and std.dev. for** $\boldsymbol{=(}\boldsymbol{1,2,3)}$ **)**

$$difference average :=\sum_{i}^{N_{g}} {i*\boldsymbol{P}}_{\boldsymbol{x-y}}(i)$$

**60. – 65. Difference Entropy (mean and std.dev. for** $\boldsymbol{=(}\boldsymbol{1,2,3)}$ **)**

$$difference entropy :=\sum_{i}^{N_{g}} \boldsymbol{P}_{\boldsymbol{x-y}}\left( i \right)*\log_{2} (\boldsymbol{P}_{\boldsymbol{x-y}}\left( i \right))$$

**66. – 71. Difference Variance (mean and std.dev. for** $\boldsymbol{=(}\boldsymbol{1,2,3)}$ **)**

$$difference variance :=\sum_{i}^{N_{g}} \left( i- \overline{P_{x-y}} \right)^{2}*\boldsymbol{P}_{\boldsymbol{x-y}}(i)$$

**72. – 77. Dissimilarity (mean and std.dev. for** $\boldsymbol{=(}\boldsymbol{1,2,3)}$ **)**

$$dissimilarity :=\sum_{i}^{N_{g}} \sum_{j}^{N_{g}} \left| i-j \right|\boldsymbol{P}(i,j)$$

**78. – 83. Energy (mean and std.dev. for** $\boldsymbol{=(}\boldsymbol{1,2,3)}$ **)**

$$energy:=\sum_{i}^{N_{g}} \sum_{j}^{N_{g}} \boldsymbol{P}\left( i,j \right)^{2}$$

**84. – 89. Entropy (mean and std.dev. for** $\boldsymbol{=(}\boldsymbol{1,2,3)}$ **)**

$$entropy :=\sum_{i}^{N_{g}} \sum_{j}^{N_{g}} \boldsymbol{P}\left( i,j \right)\log_{2} \left[ \boldsymbol{P}\left( i,j \right) \right]$$

**90. – 95. Harralick Correlation (mean and std.dev. for** $\boldsymbol{=(}\boldsymbol{1,2,3)}$ **)**

$$harralick correlation :=\frac{1}{\sigma_{x}}\left[ \sum_{i}^{N_{g}} \sum_{j}^{N_{g}} i*j*\boldsymbol{P}\left( i,j \right)-\mu_{x} \right]$$

**96. – 101. Inverse Difference (Homogeneity 1) (mean and std.dev. for** $\boldsymbol{=(}\boldsymbol{1,2,3)}$ **)**

$$inverse difference :=\sum_{i}^{N_{g}} \sum_{j}^{N_{g}} \frac{\boldsymbol{P}(i,j)}{1+ \left| i-j \right|}$$

**102. – 107. Inverse Difference Moment (Homogeneity 2, IDM) (mean and std.dev. for** $\boldsymbol{\delta}\boldsymbol{=(}\boldsymbol{1,2,3)}$**)**

$$IDM :=\sum_{i}^{N_{g}} \sum_{j}^{N_{g}} \frac{\boldsymbol{P}(i,j)}{1+\left( i-j \right)^{2}}$$

**108. – 113. Inverse Difference Moment Normalized (IDMN) (mean and std.dev. for** $\boldsymbol{=(}\boldsymbol{1,2,3)}$ **)**

$$IDMN :=\frac{1}{N^{2}}\sum_{i}^{N_{g}} \sum_{j}^{N_{g}} \frac{\boldsymbol{P}(i,j)}{1+\left( i-j \right)^{2}}$$

**114. – 119. Inverse Difference Normalized (IDN) (mean and std.dev. for** $\boldsymbol{=(}\boldsymbol{1,2,3)}$ **)**

$$IDN :=\frac{1}{N}\sum_{i}^{N_{g}} \sum_{j}^{N_{g}} \frac{\boldsymbol{P}(i,j)}{1+ \left| i-j \right|}$$

**120. – 125. Inverse Variance (mean and std.dev. for** $\boldsymbol{=(}\boldsymbol{1,2,3)}$ **)**

$$inverse variance :=\sum_{i}^{N_{g}} \sum_{j}^{N_{g}} \frac{\boldsymbol{P}(i,j)}{\left( i-j \right)^{2}}\boldsymbol{;}i \neq j$$

**126. – 131. Maximum Probability (mean and std.dev. for** $\boldsymbol{=(}\boldsymbol{1,2,3)}$ **)**

$$maximum probability :=\max\{\boldsymbol{P}\left( i,j \right)\}$$

**132. – 137. Sum Average (mean and std.dev. for** $\boldsymbol{=(}\boldsymbol{1,2,3)}$ **)**

$$sum average:=\sum_{i}^{{2N}_{g}} {i*\boldsymbol{P}}_{\boldsymbol{x+y}}\left( i \right)$$

**138. – 143. Sum Entropy (mean and std.dev. for** $\boldsymbol{=(}\boldsymbol{1,2,3)}$ **)**

$$sum entropy :=\sum_{i}^{{2N}_{g}} \boldsymbol{P}_{\boldsymbol{x+y}}\left( i \right)*\log_{2} (\boldsymbol{P}_{\boldsymbol{x+y}}\left( i \right))$$

**144. – 149. Sum Variance (mean and std.dev. for** $\boldsymbol{=(}\boldsymbol{1,2,3)}$ **)**

$$sum variance :=\sum_{i}^{{2N}_{g}} \left( i- \overline{P_{x+y}} \right)^{2}*\boldsymbol{P}_{\boldsymbol{x+y}}(i)$$

**150. – 155. Variance (mean and std.dev. for** $\boldsymbol{=(}\boldsymbol{1,2,3)}$ **)**

$$variance :=\sum_{i}^{N_{g}} \sum_{j}^{N_{g}} \left( i-\mu\right)^{2}\boldsymbol{P}(i,j)$$

**Gray-level run-length based texture features**

The gray level run length matrix (GLRLM) $\boldsymbol{P}$ summarizes how fractionated (heterogeneous) or homogeneous the gray value distribution within the ROI is. Each element $(k,l)$ describes the number of runs with $l$consecutive voxels within a gray level bin $k$ in a given direction$\theta$. The gray values are binned into $N_{g}$different bins to avoid short bins due to noise. The following example depicts the relationship of image I and corresponding GLRLM in horizontal direction:

| I= | 2 | 4 | 1 | 1 | 2 |  | GLRLM = | 3 | 2 | 0 | 0 | 0 |
| --- | --- | --- | --- | --- | --- | --- | --- | --- | --- | --- | --- | --- |
|  | 1 | 1 | 5 | 5 | 5 |  |  | 4 | 0 | 1 | 0 | 0 |
|  | 2 | 3 | 4 | 2 | 3 |  |  | 4 | 0 | 0 | 0 | 0 |
|  | 1 | 2 | 2 | 2 | 1 |  |  | 2 | 0 | 0 | 0 | 0 |
|  | 5 | 3 | 1 | 3 | 5 |  |  | 2 | 0 | 1 | 0 | 0 |

GLRLM -based features were calculated in 3D, reporting the mean and standard deviation of the features based on all possible 13 directions.

Definitions:

- $\boldsymbol{P}_{\boldsymbol{\theta}}\left( k,l \right)=\boldsymbol{P}\left( k,l \right):$Number of runs with gray value $k$ and length 𝑙 in direction $\theta$
- $N_{g}$: Number of discrete gray values
- $N_{r}$: Number of discrete run lengths
- $N_{run}$: Number of different runs
- $N_{p}$: Number of voxels in ROI

**156. – 157. Gray level nonuniformity (GLN) (mean and std.dev.)**

$$GLN:= \frac{1}{N_{run}}\sum_{k}^{N_{g}} \left[ \sum_{l}^{N_{r}} \boldsymbol{P}\left( k,l \right) \right]^{2}$$

**158. – 159. High gray level run emphasis (HGLRE) (mean and std.dev.)**

$$HGLRE:= \frac{1}{N_{run}}\sum_{k}^{N_{g}} \sum_{l}^{N_{r}} k^{2}\boldsymbol{*P}(k,l)$$

**160. – 161. Long run emphasis (LRE) (mean and std.dev.)**

$$LRE := \frac{1}{N_{run}}\sum_{k}^{N_{g}} \sum_{l}^{N_{r}} l^{2}*\boldsymbol{P}(k,l)$$

**162. – 163. Long run high gray level emphasis (LRHGLE) (mean and std.dev.)**

$$LRHGLE := \frac{1}{N_{run}}\sum_{k}^{N_{g}} \sum_{l}^{N_{r}} k^{2}l^{2}*\boldsymbol{P}(k,l)$$

**164. – 165. Long run low gray level emphasis (LRLGLE) (mean and std.dev.)**

$$LRLGLE := \frac{1}{N_{run}}\sum_{k}^{N_{g}} \sum_{l}^{N_{r}} \frac{l^{2}}{k^{2}}\boldsymbol{P}(k,l)$$

**166. – 167. Low gray level run emphasis (LGLRE) (mean and std.dev.)**

$$LGLRE:= \frac{1}{N_{run}}\sum_{k}^{N_{g}} \sum_{l}^{N_{r}} \frac{1}{k^{2}}\boldsymbol{P}(k,l)$$

**168. – 169. Number of runs (mean and std.dev.)**

$$number of runs=N_{runs}:= \sum_{k}^{N_{g}} \sum_{l}^{N_{r}} \boldsymbol{P}(k,l)$$

**170. – 171. Run length nonuniformity (RLN) (mean and std.dev.)**

$$RLN := \frac{1}{N_{run}}\sum_{l}^{N_{r}} \left[ \sum_{k}^{N_{g}} \boldsymbol{P}\left( k,l \right) \right]^{2}$$

**172. – 173. Run percentage (RP) (mean and std.dev.)**

$$RP:= \frac{N_{run}}{N_{p}}$$

**174. – 175. Short run emphasis (SRE) (mean and std.dev.)**

$$SRE:=\frac{1}{N_{run}}\sum_{k}^{N_{g}} \sum_{l}^{N_{r}} \frac{1}{l^{2}}*\boldsymbol{P}(k,l)$$

**176. – 177. Short run high gray level emphasis (SRHGLE) (mean and std.dev.)**

$$SRHGLE:= \frac{1}{N_{run}}\sum_{k}^{N_{g}} \sum_{l}^{N_{r}} \frac{k^{2}}{l^{2}}\boldsymbol{P}(k,l)$$

**178. – 179. Short run low gray level emphasis (SRLGLE) (mean and std.dev.)**

$$SRLGLE:= \frac{1}{N_{run}}\sum_{k}^{N_{g}} \sum_{l}^{N_{r}} \frac{1}{{k^{2}l}^{2}}\boldsymbol{P}(k,l)$$

***
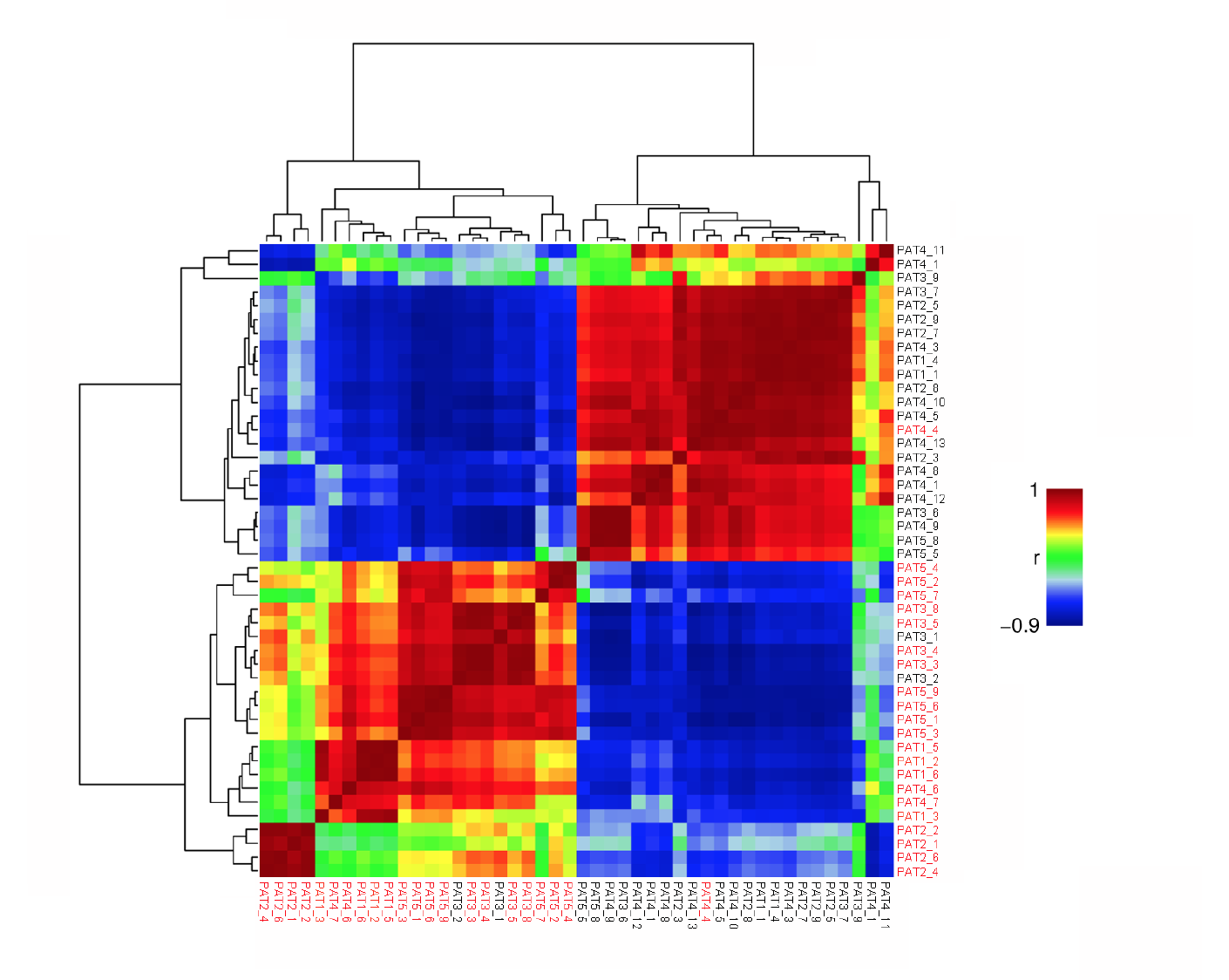
***

**Figure S1. Pairwise correlation between patient samples.** Heatmap representing pairwise correlation between patient samples based on the genome-wide methylation data. All 400 metagenes are shown. Samples highlighted in red represent samples harboring highly significant CNAs.

***Figure S2. Patient 3.***

***
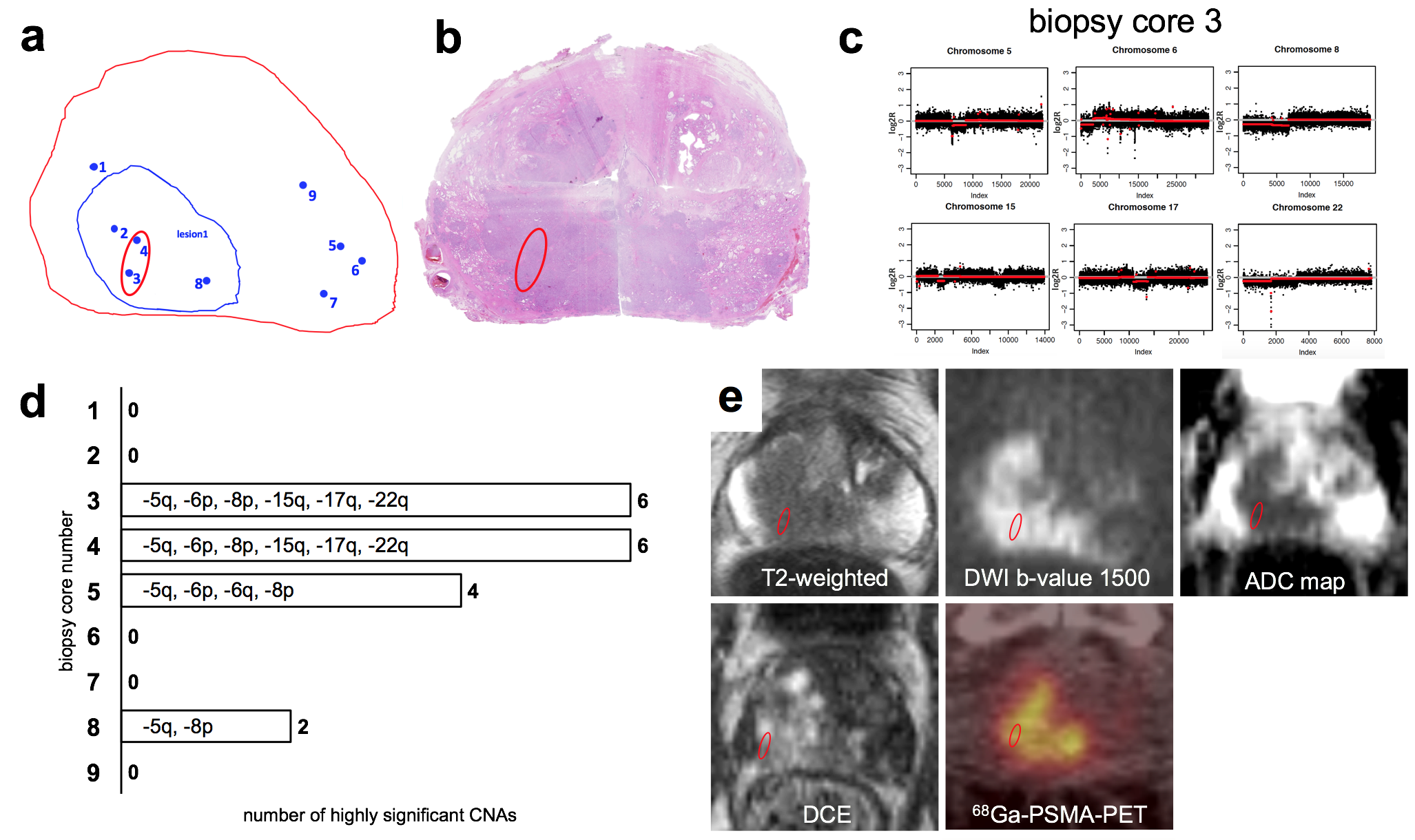
***

***Figure S3. Patient 4.***

***
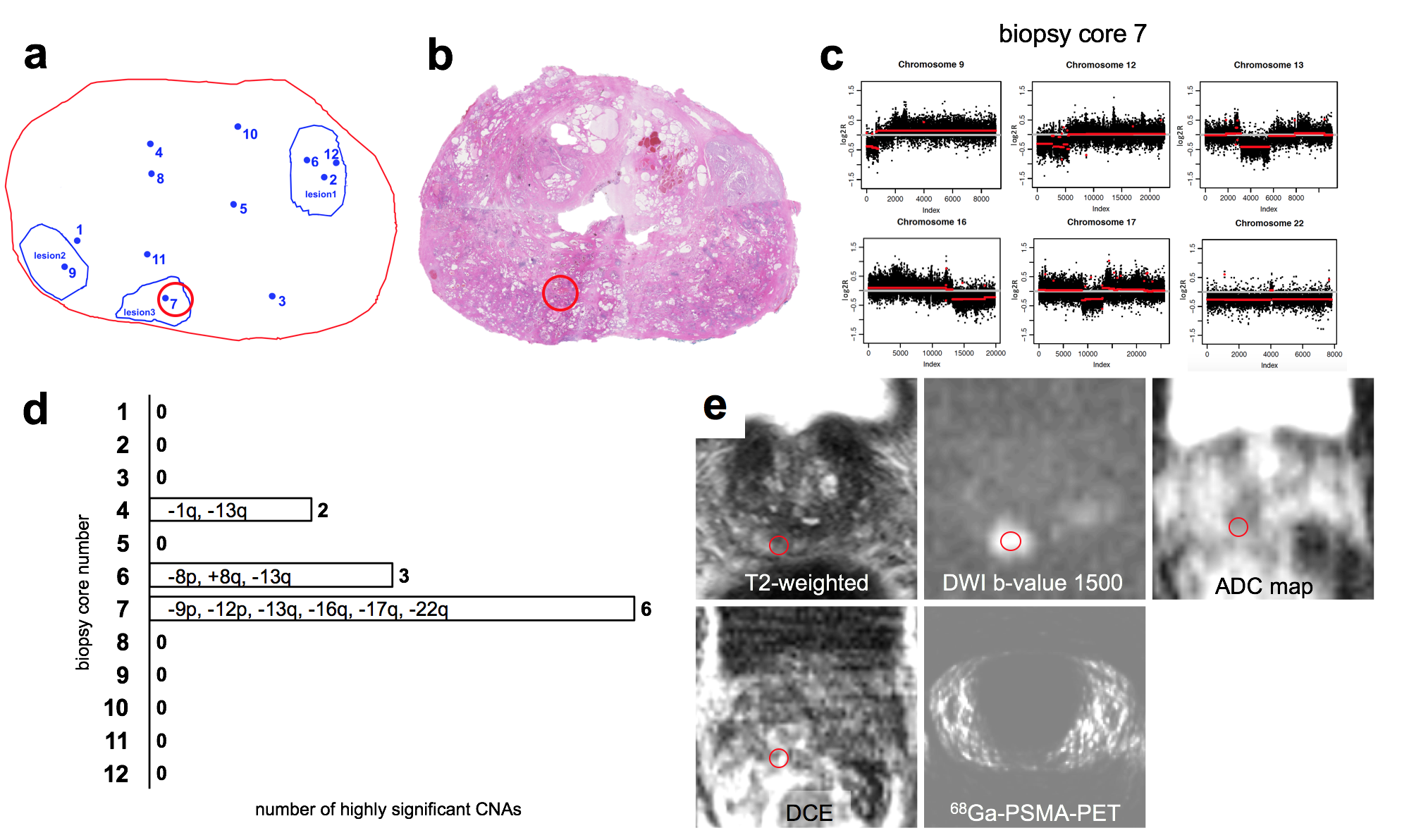
***

***
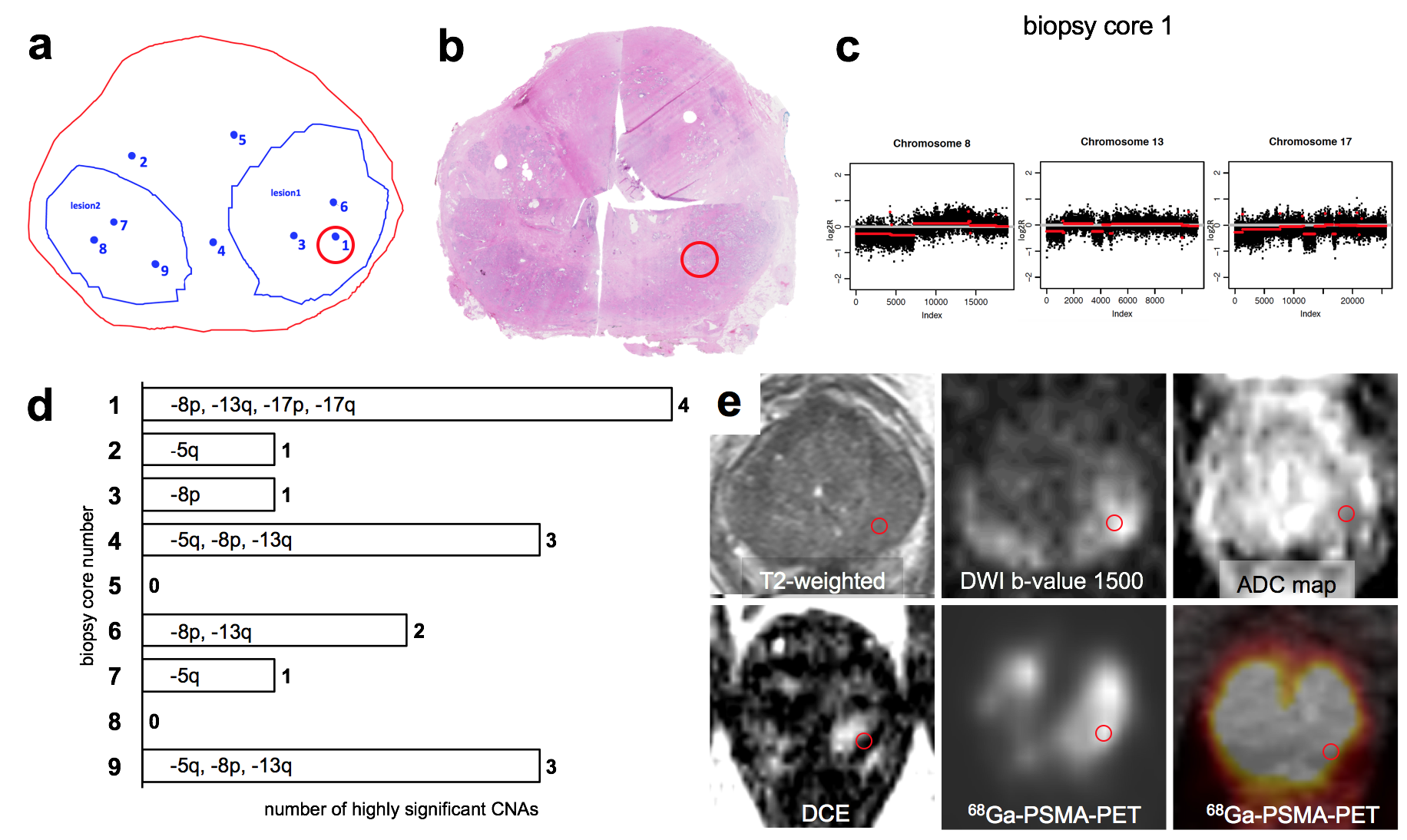
Figure S4. Patient 5.***

**Figure S2-4. Correlation between genomic index lesion and imaging parameters.**

Synopsis of (a) projection map of MRI/TRUS fusion biopsy of patient 3, 4, and 5. The prostate margin is shown in red, pre-biopsy mpMRI suspicious lesions in blue and individual biopsy cores that were sent for genomic testing as blue dots. Biopsies were performed transperineally and lesions and biopsy trajectories are projected along the base-apex direction. Genomic index lesions are encircled in red; (b) virtual whole mount of the prostatectomy specimen with red circle matching the genomic index lesion by visual comparison; (c) CNA profiles of genomic index lesions; (d) overview of CNAs of all biopsies harboring highly significant alterations; (e) mpMRI components (T2w, DWI b-value = 1500 s/mm^2^, ADC map and early arterial phase of DCE-MRI) and ^68^Ga-PSMA PET/CT are shown. The visually matched location of the genomic index lesion on individual components is superimposed on the axial slices centered on the mpMRI lesion used during MRI/TRUS biopsy.
